# Role of Functional Groups in the Monomer Molecule on the Radical Polymerization in the Presence of Graphene Oxide. Polymerization of Hydroxyethyl Acrylate under Isothermal and Non-Isothermal Conditions

**DOI:** 10.3390/molecules27020345

**Published:** 2022-01-06

**Authors:** Ioannis S. Tsagkalias, Dimitrios S. Achilias

**Affiliations:** Lab of Polymer and Colors Chemistry and Technology, Department of Chemistry, Aristotle University of Thessaloniki, 54124 Thessaloniki, Greece; itsagkal@chem.auth.gr

**Keywords:** polymerization kinetics, graphene oxide, isoconversional methods, hydroxyethyl acrylate, polymer nanocomposites

## Abstract

Functional groups in a monomer molecule usually play an important role during polymerization by enhancing or decreasing the reaction rate due to the possible formation of side bonds. The situation becomes more complicated when polymerization takes place in the presence of graphene oxide since it also includes functional groups in its surface. Aiming to explore the role of functional groups on polymerization rate, the in situ bulk radical polymerization of hydroxyethyl acrylate (HEA) in the presence or not of graphene oxide was investigated. Differential scanning calorimetry was used to continuously record the reaction rate under both isothermal and non-isothermal conditions. Simple kinetic models and isoconversional analysis were used to estimate the variation of the overall activation energy with the monomer conversion. It was found that during isothermal experiments, the formation of both inter- and intra-chain hydrogen bonds between the monomer and polymer molecules results in slower polymerization of neat HEA with higher overall activation energy compared to that estimated in the presence of GO. The presence of GO results in a dissociation of hydrogen bonds between monomer and polymer molecules and, thus, to higher reaction rates. Isoconversional methods employed during non-isothermal experiments revealed that the presence of GO results in higher overall activation energy due to the reaction of more functional groups on the surface of GO with the hydroxyl and carbonyl groups of the monomer and polymer molecules, together with the reaction of primary initiator radicals with the surface hydroxyl groups in GO.

## 1. Introduction

Graphene is a single-atomic, two-dimensional layer of sp^2^ hybridized carbon atoms arranged in a honeycomb lattice. It has recently attracted enormous research interest due to its exceptional micromechanical, electrical, thermal, and optical properties [1,2]. Graphene has an extremely high elastic modulus, E ≈ 1 TPa and ultimate strength, σ∼ 130 GPa. Adding highly exfoliated carbon layers can significantly alter the mechanical and electrical properties of polymers at extremely small loadings [3]. These properties make graphene a very important additive for the development of functional graphene-reinforced polymer composites with improved properties. The greatest scientific interest has been found in the synthesis of nanocomposites of graphene or graphene oxide with polyaniline for the production of high performance supercapacitors with enhanced electrical conductivity [3,4,5,6,7,8,9,10]. New graphene oxide-based materials were used as nanocatalysts for the preparation of xanthene derivatives including fur-imine-functionalized GO-immobilized copper oxide nanoparticles [11,12,13].

Graphene can be obtained from the exfoliation of graphite sheets. However, it is easier to obtain graphene oxide (GO) sheets through the exfoliation of graphite oxide. The latter can be produced by the oxidation of graphite and consists of many functional, oxygen-containing groups, such as epoxy, carboxyl, and hydroxyl placed either in the basal planes or the edges [2,14] Such functional groups provide graphite oxide with hydrophilicity and weaken the van der Waals forces between layers. Thus, graphite oxides can be readily dispersed in aqueous media to form colloidal suspensions, which facilitate the exfoliation of layered graphite oxide into GO sheets via sonication or stirring [15].

**Figure 1:**
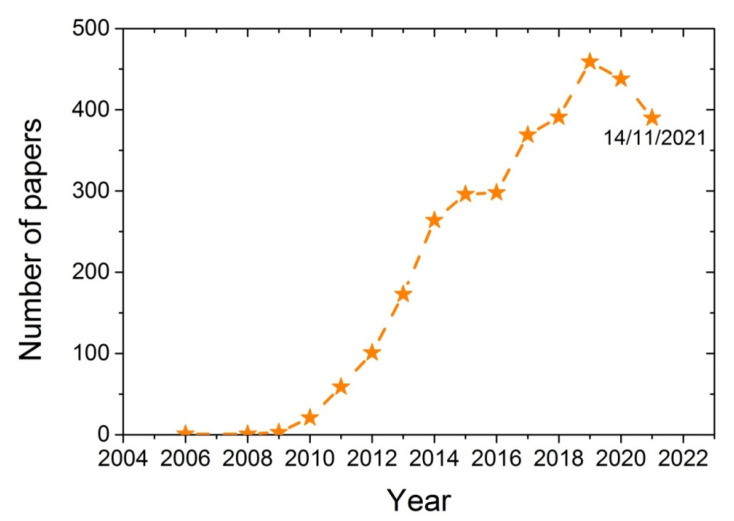
Number of papers published from 2006 to 2021 obtained from the database SCOPUS using the keywords Graphene oxide and polymerization.

Various techniques have been developed for the synthesis of polymer-based nano-composites, including melt blending, solution casting, and in situ polymerization [16,17,18]. During melt mixing, the additive is blended with the polymer when it is in the melt state and usually takes place in an extruder at temperatures above the melting point of the polymer. Although good mixing is achieved, sometimes possible chain degradation takes place, resulting in a polymer with a lower degree of polymerization and higher dispersity of its chain length distribution. Such problems are avoided with the solution casting technique, though this usually requires solvents that have to be separated and recycled and are sometimes harmful. In the in situ polymerization technique, the polymer nanocomposite is formed in situ, starting from a good dispersion of the additive in the monomer, which also ensures a good dispersion of the nanoadditive in the polymer matrix. The in situ polymerization technique for the production of nanocomposites based on several polymers and GO was also the subject of several research works conducted by our group [19,20,21,22,23]. The increasing research interest of the scientific community on the characteristics of the reaction and the properties of the nanocomposites produced when using GO during polymerization can be seen in Figure 1. From a search on the scientific database SCOPUS using the keywords Graphene Oxide and Polymerization shows that a few years ago (i.e., in 2008/2009), the number of papers published on this subject was only 1 to 3. This number recently has increased to more than 400.Therefore, it seems that using GO during polymerization is a topic of great and growing scientific interest.

In addition to polyaniline reported previously, several other polymers have been investigated for the synthesis of nanocomposites with graphene oxide. In our previous publications, poly(methyl methacrylate), PMMA, polystyrene, PS and poly(n-butyl methacrylate), and PBMA-based graphene oxide nanocomposites were studied [19,20,21,22,23].

Radical polymerization normally concerns vinyl monomers with at least one double bond in their structure. Polymerization of these vinyl monomers leads to a significant heat release due to the addition reaction to the double bond. Among other thermochemical methods, the most commonly employed for measuring the polymerization kinetics is differential scanning calorimetry (DSC) where the output signal is proportional to the rate of heat production. DSC is a very sensitive and exact technique for measuring the polymerization rate as a function of either time (isothermal mode) or temperature (non-isothermal mode), by recording the rate of heat released from the polymerizing sample, which is assumed to be proportional to the reaction rate. It offers the benefit of continuously monitoring the variation of the reaction rate, which allows the estimation and identification of the specific phenomena taking place during polymerization (such as diffusion control, as well as chemical reactions). This is very important since in other techniques for measuring the polymerization conversion (e.g., gravimetry, FTIR, etc.), only discrete experimental data are gathered. DSC measurements can be easily accomplished in a variety of experimental conditions and monomer(s) chemical structure. The latter is significant, especially when polymerization leading to crosslinked structures is investigated, where other techniques that require the dissolution of the formed polymer often fail. Use of DSC in the investigation of the isothermal bulk polymerization of a variety of methacrylate monomers, including the well-studied 2-hydroxy ethyl methacrylate (HEMA), as well as the curing of epoxy resins, has been reported in the references [24,25,26,27,28,29,30,31]. The reason for the use of DSC is that the variation in the reaction rate with time can be measured continuously during the whole reaction. In addition, despite the high amount of heat released during polymerization due to the reaction exotherm, isothermal conditions can be achieved. Polymerization enthalpy can be recorded either as a function of time in isothermal conditions or as a function of temperature in non-isothermal experiments at different heating rates. The latter have been used by isoconversional approaches to estimate the overall variation of the effective activation energy with the conversion [32]. However, investigations on the simulation of polymerization kinetics of functional acrylic monomers using isoconversional methods are rather rare.

The objective of this article is to implement the methodology employed previously for HEMA [31], for a different acrylic, this time, monomer, namely 2-hydroxyethyl acrylate (HEA). Similar to HEMA, this acrylate displays hydrogen bonding responsible for such properties as its high boiling point and water solubility. Poly(2-hydroxyethylacrylate) (PHEA) (alone or as a copolymer) has been widely used as an adhesive for binding agents for a variety of uses. PHEA finds applications in automotive top coatings, architectural coatings, photocure resins, and adhesives. Globally, about half of the HEA produced is used in the production of acrylic enamels for the automotive industry, where a clear topcoat is applied to a pigmented base coat to increase corrosion protection and durability. In this study, for the first time, the polymerization kinetic of HEA in the presence or not of graphene oxide is investigated. DSC results were treated with mechanistic or isoconversional methods to estimate the overall polymerization activation energy for both neat HEA and HEA with GO nanocomposites under isothermal and non-isothermal conditions. The purpose was to investigate the synchronous effect of functional groups on the monomer molecule and the GO surface on the polymerization kinetics. In this way, possible reaction enhancement or deceleration are explored.

## 2. Results

### 2.1. Characterization of the Materials Prepared

**Figure 2:**
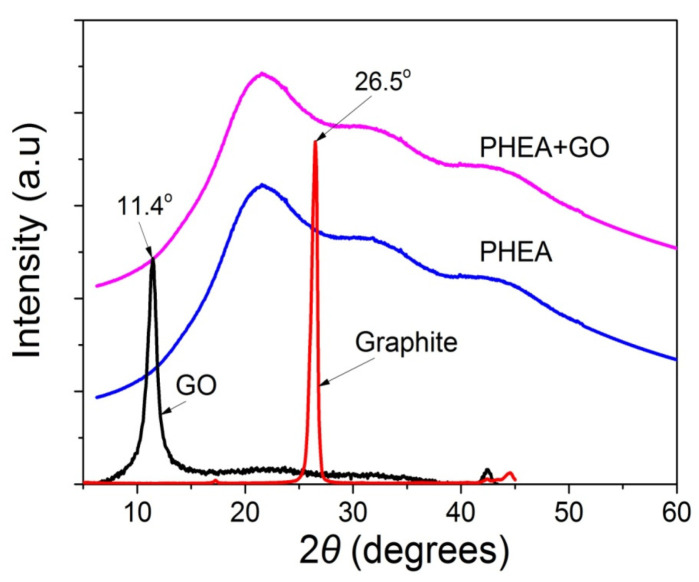
X-ray diffraction patterns of graphite, GO, neat PHEA and PHEA+GO.

In order to identify the oxidation of graphite to graphite oxide and then its exfoliation to graphene oxide, as well as the presence of GO in the polymer matrix, XRD measurements were carried out for graphite, GO, neat PHEA, and the nanocomposites of PHEA and GO. From the XRD spectra shown in Figure 2, graphite shows a sharp peak at 26.5°. When it is transformed to graphite oxide this peak is shifted to 11.4°. This means that graphite has successfully oxidized to graphite oxide, and that the latter has been exfoliated to graphene oxide during ultrasonication. In neat PHEA, a broad distribution appears denoting the amorphous structure of the polymer. When GO was incorporated into the polymer matrix, the same spectra was recorded. Neither of the characteristic sharp peaks of graphite at 26.5° or graphite oxide at 11.4° appear. This is an indication that graphite oxide has been exfoliated into graphene oxide during the reaction, but, also, it could be attributed to the very low amount of GO used.

**Figure 3:**
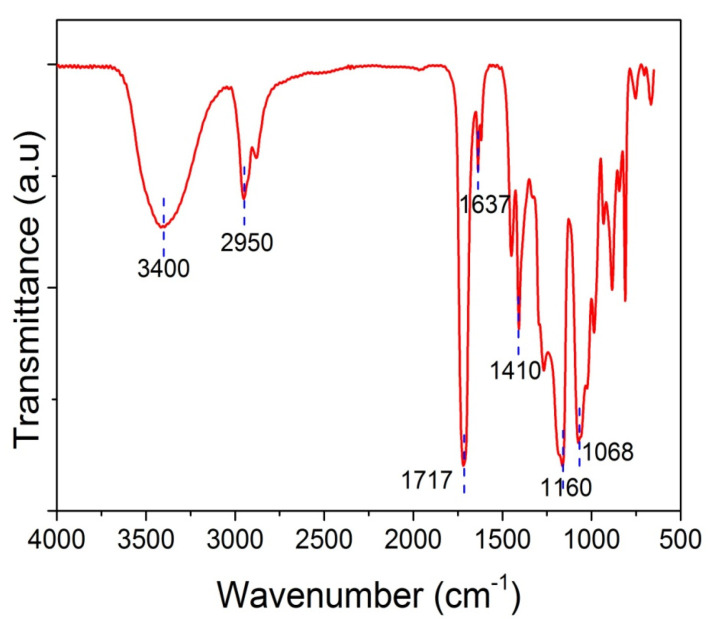
FTIR-ATR spectra of neat PHEMA.

Furthermore, spectroscopic studies were realized in order to corroborate the chemical structure of polymer-based nanocomposites via the identification of specific functional groups. Possible physicochemical interactions between GO and the PHEA hydrogel matrix were recorded using FTIR-ATR spectroscopy. Figure 3 shows the FTIR-ATR spectra of neat PHEA. Specific details of the spectra of the PHEA and GO nanocomposites are presented in Section 3. The spectrum of neat PHEA shows a sharp peak at 1710–1720 cm^−1^, which corresponds to the carbonyl bond, C=O stretching vibrations, and, in particular, to free C=O groups. Hydrogen-bonded carbonyl groups appear as a peak at 1637 cm^−1^ [33]. A very broad peak appears at 3400 cm^−1^, attributed to O-H stretching, whereas the band at 2950 cm^−1^ corresponds to the stretching vibration of the aliphatic CH_2_. The peak at 1410 cm^−1^ corresponds to bending C–O–H symmetrical deformation. The peak at 1160 cm^−1^ is attributed to stretching of the ester CO–OR group and the peak at 1068 cm^−1^ to the stretching of C–OH.

### 2.2. Isothermal Polymerization Experiments

It is well known that radical polymerization takes place mainly through three elementary reactions: initiation, propagation, and termination. Initially, the initiator is fragmented into primary radicals (benzoyloxy or phenyl in the case of the benzoyl peroxide initiator used in this investigation) by the cleavage of weak bonds, under the effect of heating or UV radiation. These primary initiator radicals find a monomer molecule to react with their double bond to start the polymerization. Following this, the radicals propagate through the addition of several monomer molecules to form macroradical chains. These, eventually find one another to terminate and produce the final polymer chains. During isothermal bulk or solution in situ polymerization experiments, it has been shown that the phenolic hydroxyl or carboxyl groups present on the GO surface may react with the initiator primary radicals by hydrogen abstraction [19,20]. The phenoxy radicals produced may scavenge another radical, resulting in the consumption of primary initiator radicals to side reactions and not resulting in the formation of macromolecular chains. Thus, the effective initiator efficiency is reduced, resulting in the reduction of the total macroradical concentration and eventually of the polymerization rate. Monomers in which this behavior has been found include methacrylates such as methyl methacrylate (MMA) or butyl methacrylate (BMA) and styrene (S) [19,20,21,22]. As a step further, in this investigation the polymerization of acrylate monomers bearing terminal hydroxyl groups (i.e., HEA) was investigated in order to examine the possible formation of side bonds of the functional groups on the surface of GO with the hydroxyl groups in the monomer molecule. Moreover, during the in situ radical polymerization of methacrylate monomers with terminal hydroxyl groups such as poly(hydroxyethyl methacrylate), PHEMA, it was found that the inclusion of nanoclays, such as montmorillonite, resulted in a slight enhancement of the polymerization rate due to the disruption of hydrogen bonding between HEMA molecules caused by the insertion of the clay platelets in between the macromolecular chains [30,31].The recent literature regarding the investigation of radicals can be found in Su et al. [34,35].

**Figure 4:**
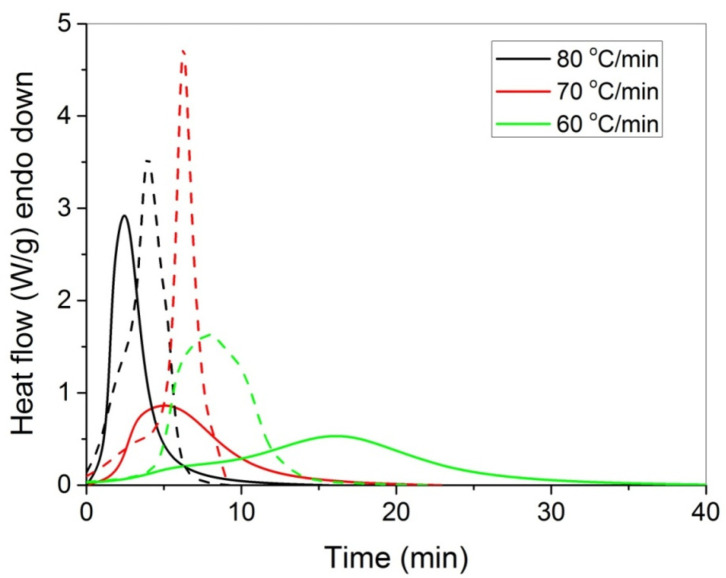
Polymerization rate, as measured by the heat release from DSC, vs. time, of HEA (continuous lines) and HEA with GO (dashed lines) at three constant reaction temperatures.

**Figure 5:**
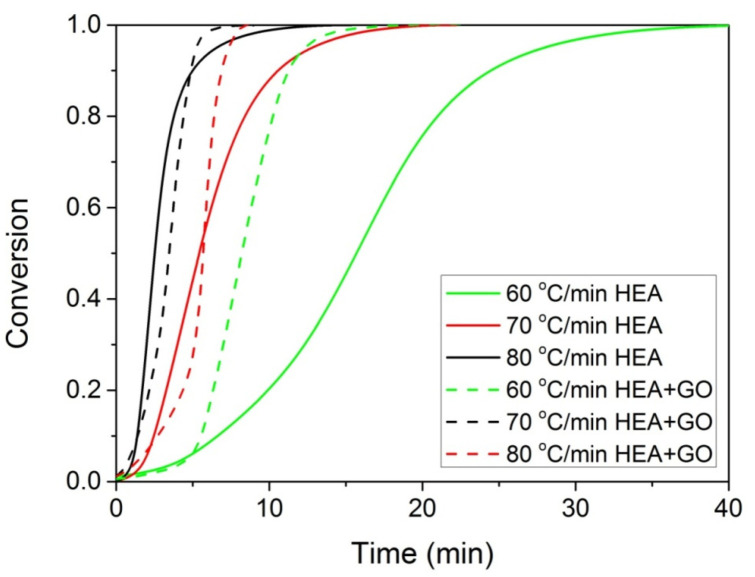
Variation of monomer conversion with time during the isothermal polymerization of either HEA or HEA with GO at three constant temperatures.

The polymerization rate, as measured by DSC, at three constant temperatures (i.e., 60, 70, and 80 °C) by the heat released vs. time, is shown in Figure 4. As expected, an increase in the reaction temperature results in an increased polymerization rate and completion of the reaction in shorter times. In the same figure, the rate of polymerization of HEA in the presence of GO is included. It can be seen that the reaction is rather fast and completes in almost 10 min at 80 °C compared to more than 1 h needed at the same conditions for the polymerization of MMA [20]. After integration of the reaction rate, the variation in the monomer conversion with time is produced and is presented in Figure 5. The polymerization of neat HEA seem to occur faster at higher temperatures compared to HEA with GO, whereas the reverse was observed at low temperatures (i.e., 60 °C).

In order to provide an explanation for the effect of GO on the polymerization kinetics, simple polymerization kinetics models were used. Accordingly, and based on the simple kinetic model for radical polymerization including initiation, propagation, chain transfer to monomer, and termination, the polymerization rate, *dX*/*dt*, assuming the steady state approximation for the total radical concentration (which has been proven to hold at low monomer conversion), is expressed as [21,30]

(1) $$\frac{dX}{dt}=\left(k_{p}+k_{trM}\right){\left(\frac{fk_{d}[I]}{k_{t}}\right)}^{1/2}(1-X)$$

where *k_p_*, *k_trM_*, *k_t_*, and *k_d_* denote the kinetic rate constants of the propagation, chain transfer to monomer, termination, and initiator decomposition reactions, respectively; *f* is the initiator efficiency; [*I*] is the initiator concentration.

Assuming that the initiator concentration remains almost constant at short reaction times (as is the case in the polymerization examined here) and all kinetic rate constants are independent of conversion, Equation (1) can be integrated to give

(2) $$X=1-\exp(-k_{eff}t)\;\mathrm{or}-\ln(1-X)=k_{eff}t$$

(3) $$k_{eff}=\left(k_{p}+k_{trM}\right){\left(\frac{fk_{d}{\left[I\right]}_{0}}{k_{t}}\right)}^{1/2}$$

The effective rate constant of common polymers, such as PMMA, *k_eff_*, can be evaluated from the available literature data on the kinetic rate constant at low conversions. However, most of the kinetic rate constants for the polymerization of HEA have not been reported in the literature. Thus, in order to have kinetic results, isoconversional methods are employed to provide the variation in the total activation energy with monomer conversion. Using the isoconversional principle and assuming Arrhenius-type dependence of all kinetic rate constants on temperature, Equation (2) can be written as

(4) $$-\ln(1-X)=A_{eff}\exp(-E_{eff}/(RT))t$$

Then, from the transformation of Equation (4), the variation in the effective overall activation energy E_eff_ with monomer conversion X can be estimated by plotting the left-hand side of Equation (5) vs. 1/T.

(5) $$\ln\left[\frac{-\ln(1-X)}{t}\right]=\ln\left(A_{eff}\right)-\frac{E_{eff}}{R}\frac{1}{T}$$

**Figure 6:**
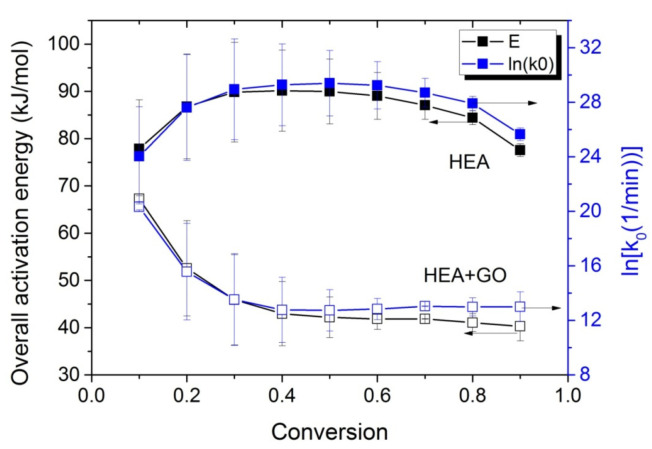
Variation in the overall polymerization activation energy and the pre-exponential factor of the kinetic rate constant with monomer conversion, estimated using the kinetic model and isoconversional methods during isothermal polymerization of HEA and HEA with GO.

At all temperatures investigated, the reaction time t at different conversion values is obtained from Figure 5 and such plots are created. From the slope of the straight line, the activation energy is estimated, whereas from the intercept the ln(A_eff_) is estimated. Such plots were created for both neat HEA and the HEA with GO nanocomposites, and the values obtained are plotted as a function of conversion in Figure 6.

The E_eff_ estimated for HEA at low monomer conversions, near 80 kJ/mol, is slightly lower than the value estimated at low monomer conversions for PHEMA (i.e., almost 90 kJ/mol [31]) and near to that estimated value at low monomer conversions for PMMA, i.e., 84 kJ/mol [30]. The activation energy seems to increase until it reaches90 k/mol at 30% conversion and remains almost constant at this value until 70% conversion, whereas it then slightly decreases to values near its original. However, in the case of the HEA with GO nanocomposites, the overall effective activation energy starts from a value near 70 kJ/mol (lower than the corresponding neat PHEA) and decreases to nearly 45 kJ/mol at 30% conversion. Afterwards, it remains almost constant at this value. The variation in the pre-exponential factor with conversion follows the tendency of the activation energy. A possible explanation is provided in the next section.

### 2.3. Non-Isothermal Experiments

**Figure 7:**
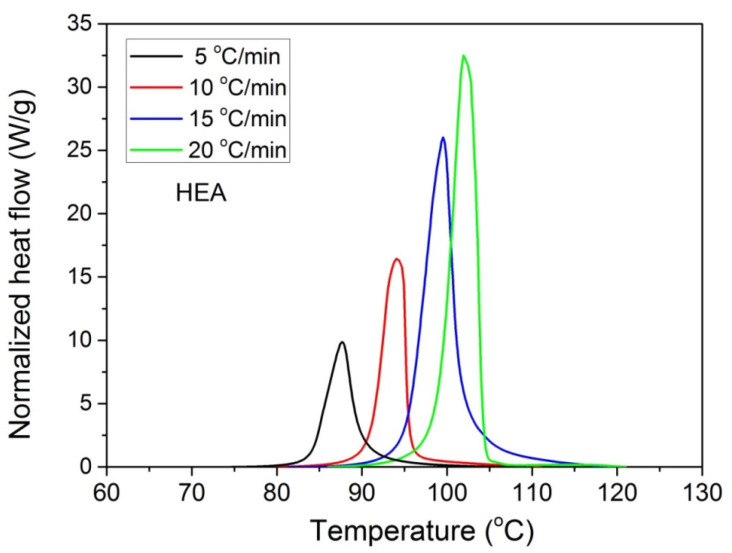
Polymerization rate, as measured by the heat release from DSC, vs. temperature, measured during non-isothermal polymerization of HEA at four heating rates.

**Figure 8:**
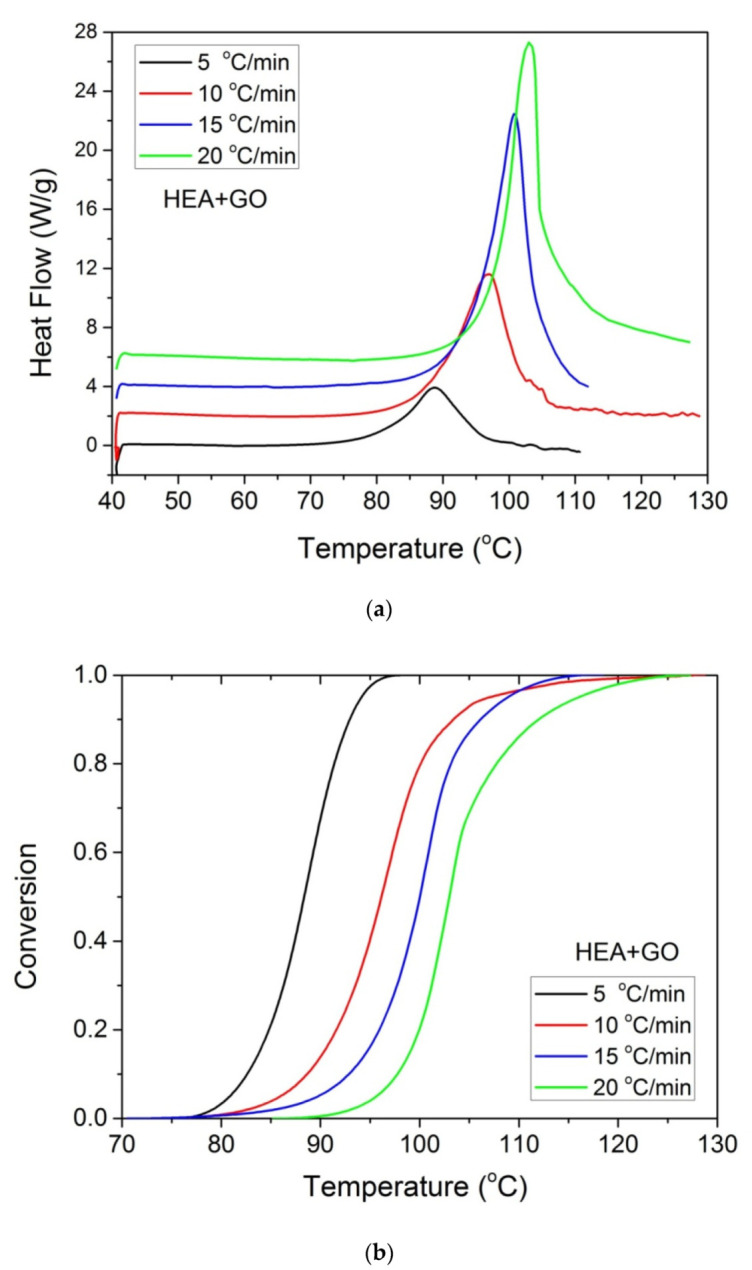
Polymerization rate, as measured by the heat release from DSC, vs. temperature (**a**) and monomer conversion (**b**), measured during non-isothermal polymerization of HEA with GO at four heating rates.

**Figure 9:**
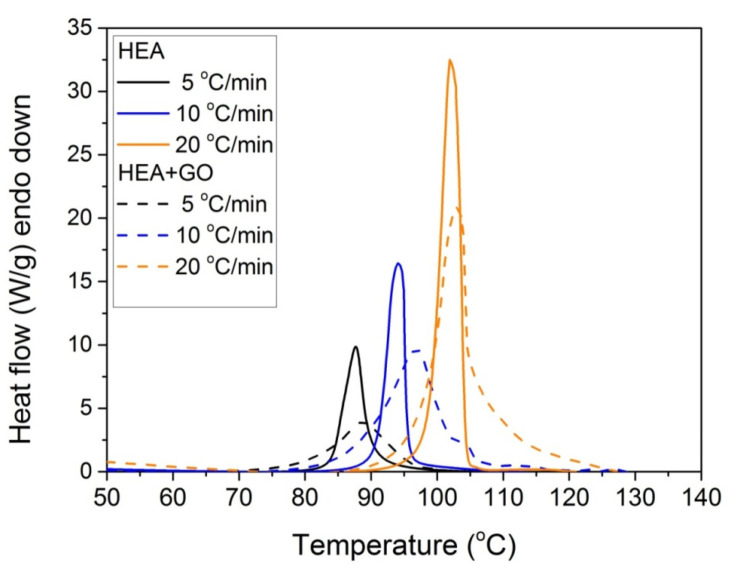
Comparative plots of the polymerization rate profile vs. temperature obtained from non-isothermal experiments of both HEA and HEA with GO at heating rates of 5, 10, and 20 °C/min.

Furthermore, it was proposed to use non-isothermal kinetic experiments in order to investigate the effect of GO on the polymerization kinetics. Therefore, additional experiments were carried out at four heating rates from 5 to 20 °C/min. Results for both neat HEA and HEA in the presence of GO polymerization appear in Figure 7 and Figure 8a, respectively. As expected, an increase in the heating rate resulted in shifting of the reaction curves to higher temperatures. From integration of the polymerization rate curves the variation in monomer conversion with temperature this time was estimated, illustrated in Figure 8b. Polymerization starts near 80 °C at the lower heating rate (5 °C/min) and ends near 95 °C. At the high heating rate of 20 °C/min, polymerization starts and ends at 95 and 110 to 120 °C. A direct comparison between the polymerization rate profiles of neat HEA and HEA with GO appears in Figure 9. This shows that all curves measured with the addition of GO are broader compared to the corresponding curves without GO. Therefore, theoretical models were again used to address the polymerization kinetics. In non-isothermal experiments, isoconversional models are usually used to estimate the variation in the overall reaction activation energy with conversion [32]. Then, the general kinetic equation is written as

(6) $$\frac{dx}{dt}=\beta \frac{dx}{dT}=k_{eff}f(x)$$

Using an Arrhenius-type expression again for the kinetic rate constant and taking the logarithm of Equation (6) we have

(7) $$\ln\left(\beta \frac{dx}{dT}\right)=\ln\left[A_{eff}f(x)\right]-\frac{E_{eff}}{R}\frac{1}{T}$$

Using the results of the polymerization rate and conversion vs. temperature shown in Figure 6, one can estimate the values of dx/dT and T at specific conversions. Therefore, Equation (7) in an isoconversional model is re-written as

(8) $$\ln{\left(\beta \frac{dx}{dT}\right)}_{X}=\ln{\left[A_{eff}f(x)\right]}_{X}-\frac{{\left(E_{eff}\right)}_{X}}{R}\frac{1}{T_{X}}$$

By plotting the left-hand side of Equation (8) as a function of 1/T, the effective activation energy can be estimated as a function of the monomer conversion x from the slope of the straight lines obtained.

**Figure 10:**
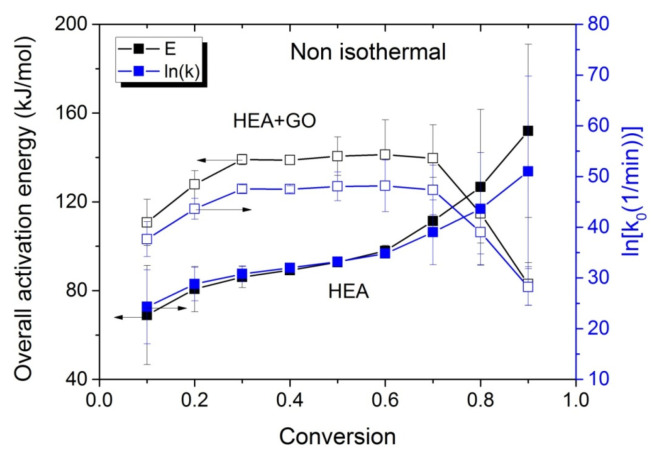
Variation in the overall polymerization activation energy and pre-exponential factor of the kinetic rate constant with monomer conversion estimated using isoconversional methods during the non-isothermal polymerization of HEA and HEA with GO.

The variations in the effective activation energy with conversion for neat HEA and HEA with GO polymerization appear in Figure 10. The picture this time is different than that observed during the isothermal experiments, meaning that the effective overall activation energy estimated when GO was added, was greater than that of neat HEA at conversions from 0.1 to 0.7. Afterwards, an increase is observed in the E_eff_ of neat HEA and a decrease in the HEA and GO polymerization. An explanation is provided in the next section, though we keep in mind that all temperatures measured during the non-isothermal experiments were always higher than the corresponding temperatures during the isothermal ones.

## 3. Discussion

During the isothermal experiments carried out at a low temperature, a possible explanation of the lower activation energy of the polymerization when GO was added is described next. It should be mentioned here that isothermal experiments at higher temperatures (i.e., 100, 110 °C) were not carried out because the whole polymerization time was very short, in the order of a few (2–3) min, and it was very difficult for this to be recorded by the instrument if one takes into consideration the equilibration time. Always, the start of the reaction was missed.

**Figure 11:**
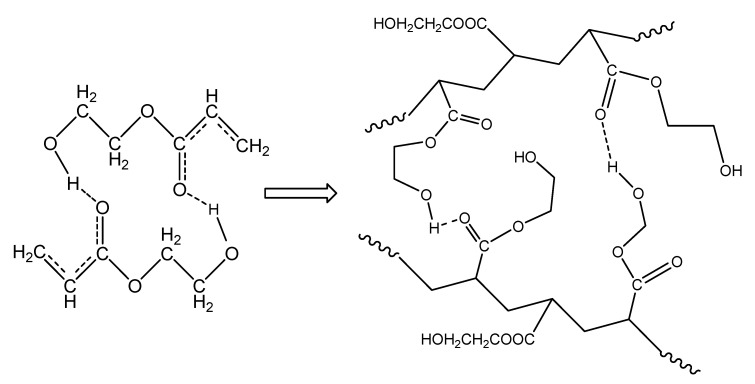
Formation of hydrogen bonds between monomer molecules during polymerization of hydroxyethyl acrylate.

The isothermal polymerization results of neat HEA can be explained in a similar way to PHEMA polymerization at the same reaction temperatures. The interpretation of these results can be carried out in terms of specific interactions and, in particular, the formation of intra- and inter-chain hydrogen bonds between the monomer and the polymer molecules. Accordingly, since the monomer, 2-hydroxyethyl acrylate, contains one hydroxyl (–OH) and one carbonyl (C=O) group on its molecule, the latter can act only as the proton acceptor, while the OH group could act as both the proton donor and acceptor [31,33]. Hydrogen bonding between the monomer hydroxyl group and carbonyl oxygen atom strengthens the positive partial charges at the carbonyl C atom and at the double bond, as shown schematically in Figure 11, leading to a significant charge transfer in the transition state of propagation [31]. During polymerization, both intra- and inter-chain hydrogen bonding between the monomer and polymer molecules may take place and, in the polymer structure, both OH---OH and C=O---HO types of hydrogen bonds can occur (Figure 11).

**Figure 12:**
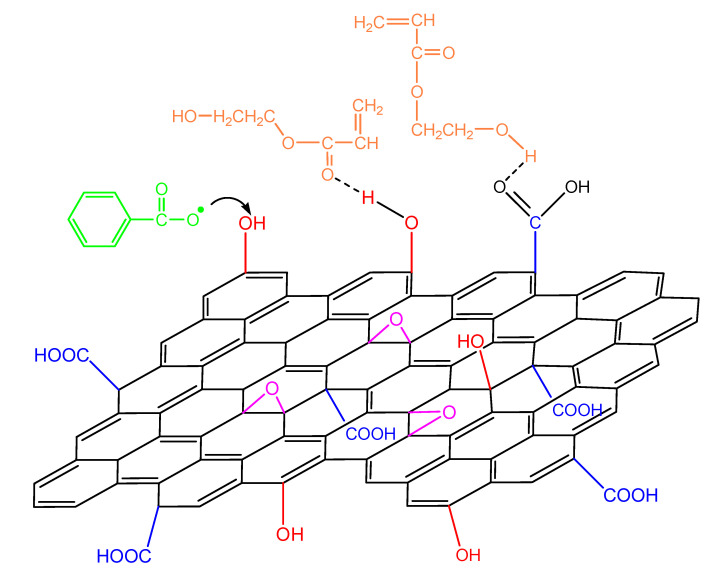
Reaction of an initiator primary radical with surface hydroxyl groups in the GO surface and hydrogen bond formation from the interaction of the functional groups on the monomer molecule and the GO surface during polymerization of hydroxyethyl acrylate.

When GO is added in the polymerizing mixture, the surface hydroxyl and carboxyl groups could have a double role: the formation of hydrogen bonds with the monomer molecule as well as reacting with the primary initiator radicals (Figure 12). The latter, as shown in our previous publications [19,20,21,22], leads to a reduction in the initiator efficiency and, as a result, to a retardation of the reaction.

**Figure 13:**
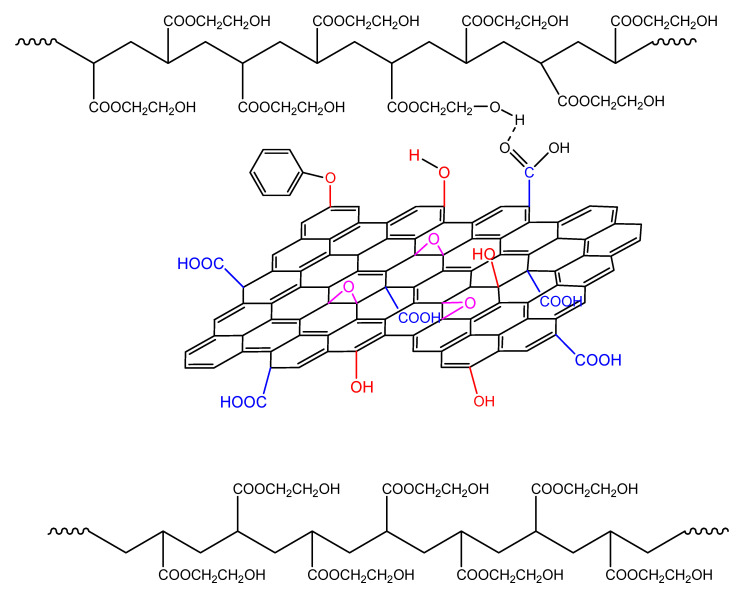
Formation of hydrogen bonds between the hydroxyl groups on the polymer chains and functional groups on the GO surface during polymerization of hydroxyethyl acrylate in the presence of GO.

During polymerization in the presence of GO it seems that the GO sheets are inserted in between the macromolecular chains. Thus, the HEA–HEA interactions with the hydrogen bonding are disrupted by the existence of the GO sheets inserted in between the monomer molecules and the macromolecular chains (Figure 13). This could result in more reactive monomers that could facilitate the reaction rate, resulting in higher kinetic rate constants and less overall activation energy.

**Figure 14:**
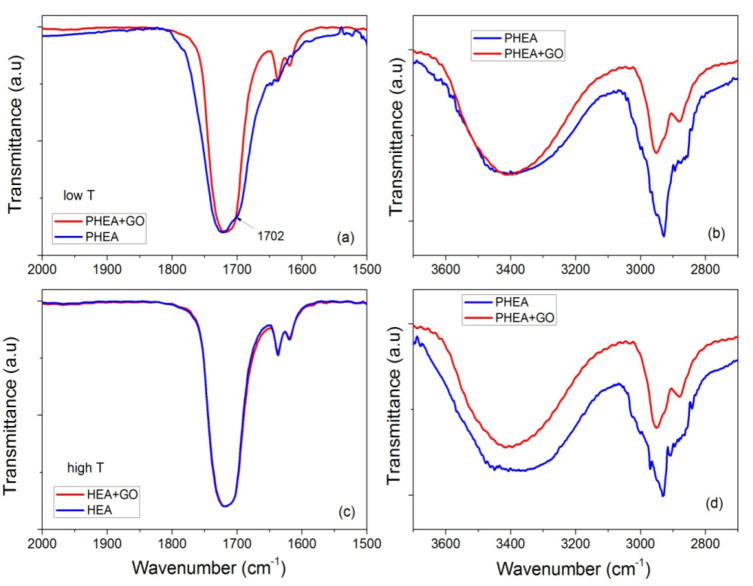
FTIR-ATR spectra focusing on the regions 2000–1500 and 3700–2700 cm^−1^, of neat PHEA and PHEA + GO obtained at low temperature (i.e., 60 °C (**a**,**b**)) or high temperature (i.e., 80 °C (**c**,**d**)).

These statements are reinforced from the FTIR measurements. Specific details of the spectra of neat PHEA and PHEA with GO nanocomposites are presented in Figure 14, focusing on the regions 2000–1500 and 3700–2700 cm^−1^. At low temperature, it is seen that the peak at 1702 cm^−1^ recorded for neat PHEA is shifted to 1717 cm^−1^ by the addition of GO (Figure 14a). According to Morita [33], the band at 1702 cm^−1^ is assigned to C=O hydrogen bonded with –OH (C=O---HO) in the PHEA chain, whereas shifting to higher wavenumbers (such as 1717 cm^−1^ in this study) is attributed to free C=O. Therefore, it seems that the presence of GO results in weakening or breaking of these hydrogen bonds and the formation of new (less in number) bonds between GO and the electron rich groups (such as hydroxyl, OH, or carbonyl, C=O) present in the hydrogel network.

Moreover, at high temperatures, monomer molecules and macroradicals have enough mobility, and their movement is not affected much by the presence of the side hydrogen bonds. Therefore, hydrogen bonding takes place at the same rate for neat PHEA and PHEA with GO. Therefore, their FTIR spectra are almost identical (Figure 14c). However, the decrease in the initiator efficiency when GO is present results in lower overall kinetic rate constant.

**Figure 15:**
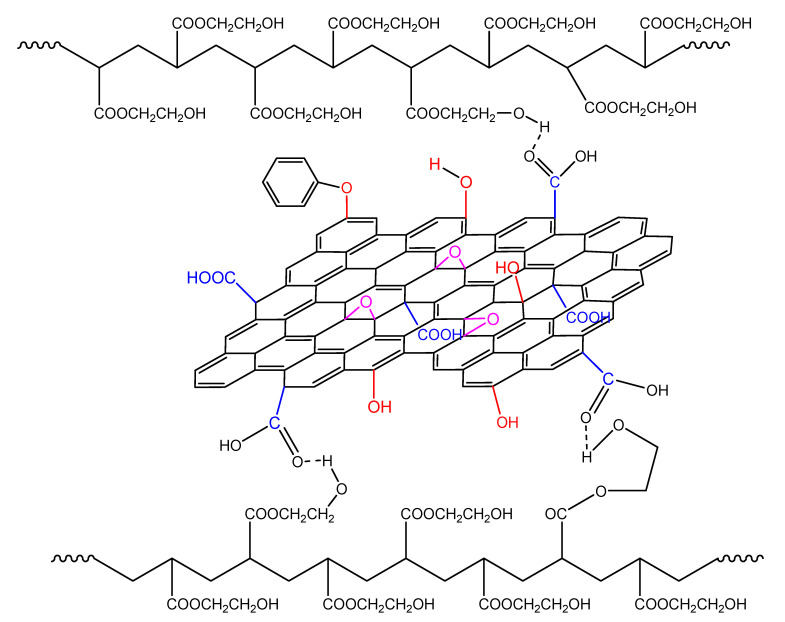
Formation of hydrogen bonds between the hydroxyl groups on the polymer chains and functional groups on the GO surface during the non-isothermal polymerization of hydroxyethyl acrylate in the presence of GO.

In non-isothermal polymerization, the reaction rate when adding GO becomes broader due to the following reasons: Initially, the higher temperatures encountered result in a higher rate of decomposition of the initiator molecules and, thus, more primary radicals that may react with the surface hydroxyl groups of GO, resulting in lower initiator efficiency and lower reaction rates. In addition, the greater reactivity due to the increased temperature result in the formation of more hydrogen bonds between the GO sheets and the HEMA groups in the macromolecular chains (Figure 15). This makes the polymerization more difficult resulting in higher overall activation energy.

## 4. Materials and Methods

### 4.1. Materials

The monomer HEA used during the experimental process had purity≥99% and was purchased from Sigma-Aldrich (Taufkirchen, Germany). In addition, the freeradical initiator, benzoyl peroxide (BPO) with a purity >97%, was provided by Fluka (Leicestershire, UK) and purified by the method of fractional recrystallization twice from methanol (Merck). Graphite powder was purchased from Sigma-Aldrich. All other chemicals used were of reagent grade.

### 4.2. Preparation of Graphite Oxide

Graphite oxide (GO) was prepared by oxidizing the graphite powder, in accordance with the Hummers method. Details can be found in our previous work [20]. Accordingly, 10 g of commercial graphite powder was dispersed in sulfuric acid (230 mL) at 0 °C. Subsequently, 30 g of potassium permanganate (KMnO_4_) was slowly added to the suspension by controlling the addition rate and maintaining the temperature below 20 °C. Following this, the reaction mixture was cooled to 2 °C. Then, the mixture was removed from the ice bath and stirred with a magnetic stirrer at room temperature for 30 min. Subsequently, 230 mL of deionized water was added, again controlling the addition rate, while temperature was kept below 20 °C. Thereafter, the mixture was resuspended under mechanical agitation for 15 min, followed by addition of 1.4 L deionized water and 100 mL of hydrogen peroxide solution (30 wt%). The mixture was allowed to stand for 24 h. The GO particles that settled at the bottom were separated from the excess liquid by decantation. The gelatinous texture material was placed in an osmotic membrane to stop the formation of precipitate BaSO_4_, which appeared during the addition of BaCl_2_ aqueous solution. The material remained in the membrane for about 8 days. Finally, the final product was obtained by freeze-drying method.

### 4.3. Preparation of the Initial Monomer/GO Mixtures

Monomers with graphite oxide were positioned for ultrasonication for one hour so there was a satisfactory colloidal dispersion of graphite oxide to the solution, while exfoliation of graphite oxide to graphene oxide started. In the final suspension, the initiator BPO 0.03 M was added and the mixture degassed by passing nitrogen and immediately used. The nanocomposites were prepared using a relative amount of GO to the monomer 0.5 wt%. Neat polymer was also synthesized under the above conditions and used as reference material.

### 4.4. Polymerization Kinetics

Polymerization was explored using the DSC, Diamond (from PerkinElmer, Waltham, MA, USA) equipped with the Pyris software for windows. Indium was chosen for the enthalpy and temperature calibration of the instrument. Polymerizations were implemented under both isothermal and non-isothermal conditions. Isothermal polymerizations were carried out at constant reaction temperatures ranging between 60 and 80 °C, whereas in non-isothermal experiments, constant heating rates were practiced varying from 5 to 20 °C min^−1^. In the isothermal experiments, the reaction temperature was listed and maintained stable (within ± 0.01 °C) throughout the conversion range. The samples, which weighted approximately 10 mg, were left unsealed and placed into the appropriate position of the instrument under nitrogen air stream. The reaction exotherm (in normalized values, W g^−1^) was documented as a function of time or temperature. The rate of heat release (d(ΔH)/dt) measured by the DSC was directly converted into the overall reaction rate (dx/dt) after the use of the following formula

(9) $$\frac{dx}{dt}=\frac{1}{\Delta H_{T}}\frac{d(\Delta H)}{dt}$$

Monomer conversion, x, can be estimated by integrating Equation (9)

(10) $$x=\int_{0}^{t}\frac{1}{\Delta H_{T}}\frac{d(\Delta H)}{dt}dt$$

where ΔH_T_ denotes the total reaction enthalpy and x the fractional conversion.

The polymerization enthalpy and conversion were estimated by integrating the area between the DSC curves and the baseline established by extrapolation from the trace produced after complete polymerization (invariable heat produced during the reaction). The residual monomer content and the total reaction enthalpy were defined by heating the sample from the polymerization temperature to 180 °C at a rate of 10 K min^−1^. The sum of enthalpies of the isothermal plus the dynamic experiment was the total reaction enthalpy. After the end of the polymerization, the pans were weighed again and a negligible loss of monomer (less than 0.2 mg) was detected only in a few experiments.

In addition, non-isothermal experiments were performed with heating rates varying from 5 to 20 °C min^−1^, and polymerization rate and monomer conversion were estimated by using methods similar to Equations (9) and (10).

### 4.5. Measurements

Fourier-Transform Infra-Red (FTIR). The chemical structure of the neat PHEA and PHEA+GO nanocomposites was confirmed by recording their IR spectra. The instrument used was the Spectrum 1 spectrophotometer from PerkinElmer with an attenuated total reflectance (ATR) device. ATR was necessary since the samples with GO were not transparent. Measurements were carried out using thin films prepared in a hot hydraulic press and spectra recorded over the range from 4000 to 600 cm^−1^ at a resolution of 2 cm^−1^, and 32 scans were averaged to reduce noise. The instrument’s software was used to identify several peaks.

X-ray diffraction. The crystalline structure of graphite, GO, as well as the prepared PHEA and GO nanocomposites were characterized using X-ray diffraction (XRD) in a Rigaku Miniflex II instrument equipped with CuKa generator (*λ* = 0.1540 nm). The XRD patterns were recorded at the range 2*θ* = 5–65° and scan speed of 2° min^−1^.

## 5. Conclusions

The in situ radical polymerization of hydroxyethyl acrylate in the presence or not of graphene oxide was studied in order to investigate possible interactions between functional groups in the monomer molecule with the hydroxyl and carbonyl groups in the surface of GO. Polymerization kinetics were studied both isothermally and non-isothermally. During isothermal polymerization, it was found that the activation energy of the polymerization taking place in the presence of GO was lower than that of neat GO. This was attributed to the formation of both intra- and inter-chain hydrogen bonds between the monomer and polymer molecules, which somehow hinder polymerization. The presence of GO results in a dissociation of hydrogen bonds between the monomer and polymer molecules resulting in higher reaction rates. Particularly at low temperatures, the presence of GO results in a higher overall kinetic rate constant, whereas the reverse holds at higher temperatures. In the latter case, hydrogen bonding takes place at almost the same rate for both neat PHEA and PHEAwithGO, but the reaction of the surface hydroxyl groups with the initiator primary radicals results in a lower overall kinetic rate constant. Isoconversional methods were used during non-isothermal experiments to estimate the variation in the overall activation energy with monomer conversion. This time the presence of GO resulted in higher overall activation energy due to the reaction of more functional groups on the surface of GO with the hydroxyl and carbonyl groups of the monomer and polymer molecules together with the reaction of the primary initiator radicals with the surface hydroxyl groups of GO.

## Data Availability

Not applicable.

## Sample Availability

Samples of the compounds are available from the authors.
